# Methodologies for establishing and validating cut-points and comparative standards in medical imaging-based body composition analysis: a scoping review protocol

**DOI:** 10.1186/s13643-026-03096-y

**Published:** 2026-04-10

**Authors:** Alanood Aljanahi, Kathryn V. Dalrymple, Eirini Dimidi, Erin Stella Sullivan

**Affiliations:** Department of Nutritional Sciences, School of Life Course & Population Sciences, Faculty of Life Sciences & Medicine, King’s College London, London, SE1 9NQ UK

**Keywords:** Body composition assessment, Diagnostic thresholds, Medical image analysis, Radiomics validation, Cut-off points, Standardisation methods, Prognostic factor, Nutritional assessment

## Abstract

**Background:**

Medical imaging-based body composition analysis (BCA) has shown promise in offering detailed, noninvasive assessments of fat, muscle, and bone, but challenges persist in establishing consistent comparative standards. Current studies reveal significant variability in methodologies, which limits comparability and clinical application. This highlights the need for a comprehensive review to explore these methodologies and address the gap in standardisation. The aim of the study is to identify and map the methodologies used in body composition imaging to establish and validate comparative standards (such as cut-points, thresholds, or normative values) and to catalogue the proposed comparative standards.

**Methods:**

This scoping review will be conducted following JBI methodology. The following eligibility criteria will be applied: Population: healthy subjects with no major comorbidities or individuals with cancer assessed using body composition imaging (BCI) and concept: methodologies for establishing BCI comparative standards and/or formally validating them against any outcome or other BCA reference standard. This scoping review will consider studies across all clinical settings. There will be no restrictions on the setting or purpose of the original study. Validation studies using BCI as the reference standard will not be included unless the comparative standard being validated is another BCI feature. The electronic databases to be searched are Ovid MEDLINE, Ovid Embase, Scopus, EBSCOhost CINAHL, Web of Science, Cochrane Library, and IEEE Xplore. Grey literature sources will not be included. Studies published in English will be considered, with no date restrictions applied. Two independent reviewers will screen all titles and abstracts, followed by full-text articles, and will undertake data extraction. Data extracted will be presented in tabular and/or diagrammatic form for comprehensive narrative synthesis.

**Discussion:**

The scoping review will summarise existing evidence on BCI. It will identify potential methodological gaps, describe current proposed thresholds or normative values, and highlight areas for further research to establish validated cut-points.

**Systematic Review Registration:**

OSF 10.17605/OSF.IO/QZMN2.

**Supplementary Information:**

The online version contains supplementary material available at 10.1186/s13643-026-03096-y.

## Background

Abnormal body composition is a hallmark of malnutrition, which can arise from age-related physiological decline, starvation-related causes, or disease-related inflammation [1, 2]. Age-related malnutrition often involves the progressive loss of muscle mass and function [3], while disease-related malnutrition, such as that seen in cancer or chronic organ failure, can involve both muscle and fat depletion [4]. With the advent of GLP-1 agonist medications, there is also a growing focus on the importance of body composition during intentional weight loss. Accurate body composition assessment (BCA) is therefore essential for identifying and managing nutritional concerns across both clinical and public health contexts [5].

While traditional nutritional assessment techniques like anthropometry and bioelectrical impedance analysis (BIA) are commonly used to infer information about body composition, they often rely on broad assumptions and do not directly measure body composition compartments; thus, they do not offer the level of accuracy needed for detailed, reliable assessments [6]. In comparison, body composition imaging (BCI) uses medical imaging-based methods for precise, consistent, and direct but noninvasive evaluation of essential body components including fat, muscle, and bone by leveraging various imaging modalities, such as computed tomography (CT), magnetic resonance imaging (MRI), positron emission tomography (PET), ultrasound, and dual-energy X-ray absorptiometry (DXA) [7].

However, despite the growing interest in BCI, the lack of standardised and validated cut-points for BCI-derived parameters remains a significant barrier to clinical implementation [8]. Research is relatively advanced in oncology, due to the availability of opportunistic CT scans, and abnormal BCI findings are associated with poorer treatment outcomes and survival [9]. Yet, even in this setting, there is no consensus on the optimal thresholds for defining low muscle mass or abnormal adiposity, and cut-points vary widely across studies, typically based on already at-risk populations, rather than normal physiology [10]. Establishing reliable, validated body composition cut-points is critical for stratifying patients, guiding treatment decisions, and improving prognostic accuracy across both cancer and broader clinical populations [8, 10, 11].

A major obstacle is the substantial heterogeneity in how diagnostic cut-points are determined. Diagnostic cut-points are defined numerical thresholds used to classify individuals into specific clinical or risk categories based on quantitative measurements [12]. Other commonly used methods to identify abnormalities in body composition include comparison to normative data or calculation of risk scores incorporating multiple risk factors. In this review, the term comparative standards will be used to encompass the broad category of (a) screening, diagnostic, or staging cut-points; (b) normative data, such as population reference curves, nomograms, or centiles; and (c) multicomponent risk scores which include at least one BCA feature.

Various methodologies have been employed to derive these thresholds, ranging from arbitrary approaches, such as tertile [13] or quartile-based divisions [14], to traditional statistical techniques, like receiver operating characteristic (ROC) analysis [12], and more advanced methods including machine learning classifiers [15] and deep learning models [16]. These methods differ in their assumptions, validation strategies, and clinical applicability and are often applied without external validation or clear justification for the chosen approach. This lack of methodological consistency in comparative standard development and validation complicates clinical translation and cross-study comparability [17]. To enable clinical adoption, harmonised protocols are needed not only for generating reproducible medical imaging measurements [18, 19] but also for establishing and validating clinically meaningful comparative standards that are robust, generalisable, and applicable across imaging modalities, devices, and clinical contexts [17, 19].

This review will map the methods used to develop and validate comparative standards for BCI in both healthy populations and cancer cohorts. In April 2025, we conducted a preliminary search across major databases such as MEDLINE, Embase, CINAHL, Scopus, Web of Science, Cochrane Database, and IEEE Xplore. This search revealed an increasing amount of literature utilising radiomics and artificial intelligence (AI) for BCI, especially in oncology, and musculoskeletal or metabolic conditions, such as type 2 diabetes, sarcopenia, and obesity. A recent systematic review and meta-analysis highlighted the effectiveness of AI-based tools, particularly deep learning models, in assessing body composition and diagnosing sarcopenia using CT imaging [20]. Several scoping and narrative reviews have focused on the role of imaging modalities [7], discussed clinical implications [21], or explored BCI applications in specific cancers [22, 23]. Despite their contributions, these reviews are often disease- or modality specific, and none systematically maps the development and validation of diagnostic cut-points.

Our preliminary review also uncovered significant methodological diversity and heterogeneity in outcome definitions and validation techniques, reflecting a fragmented evidence base. Additionally, searches conducted in the PROSPERO and JBI Evidence Synthesis databases confirmed the absence of any ongoing or completed reviews specifically addressing the methodologies used to establish and validate comparative standards for BCI. These findings underscore a critical gap in the literature and justify the need for this scoping review, which will comprehensively explore and categorise the methodological landscape in this evolving area. A scoping review was chosen for this topic due to the heterogeneity in relevant study types and methodologies [24]. Scoping reviews can accommodate diverse study designs and analytical frameworks. This approach will allow the identification of common practices, challenges, and gaps in methodology that may inform the development of standardised protocols and future research priorities.

## Methods

This scoping review protocol was preregistered on Open Science Framework (OSF) before the final search was conducted and is available at 10.17605/OSF.IO/QZMN2. The proposed scoping review will be conducted in accordance with the Joanna Briggs Institute (JBI) methodology for scoping reviews and following the Preferred Reporting Items for Systematic reviews and Meta-Analyses extension for Scoping Reviews (PRISMA-ScR) [24, 25]. A completed PRISMA-P checklist is provided as Additional File 1.

### Aims and objectives

The overall aim of this scoping review is to identify the methodologies used to establish and validate comparative standards for BCI, to map the proposed comparative standards, and to identify the clinical outcomes reported in the included studies.

### Review questions

1. What methodologies are used in medical BCI research to establish comparative standards?
2. What methodologies are used in BCI research to validate comparative standards?
3. What comparative standards have been proposed?
4. Within the included studies, what clinical outcomes have been investigated in relation to the proposed or validated comparative standards?

### Eligibility criteria

#### Population

Studies involving healthy participants (no major comorbidities, e.g. cardiovascular, metabolic, or malignant conditions) or participants with cancer, evaluated for body composition using medical imaging analysis, will be considered for this scoping review. There are no restrictions on age, sex, location, timepoint of measurements, or setting.

#### Concept

This scoping review will include studies that report on the establishment of BCI comparative standards for use beyond a single study or setting. Studies will be excluded if they do not report any BCI features, do not propose a new comparative standard, do not attempt to validate an existing one against another outcome or body composition reference standard, or do not describe their methodology in sufficient detail for replication, e.g. apparently arbitrary choice of cut-points. Studies establishing comparative standards including at least one BCI feature will be eligible. Studies which do not propose a new comparative standard generalisable beyond the scope of their study, or only validate an existing cut-point, will be excluded. Validation studies using BCI as the reference standard will be excluded, unless the comparative standard being validated established is also a BCI feature.

#### Context

This scoping review will consider studies across all contexts, provided they propose a BCI comparative standard for use beyond their own research and the methodology is sufficiently described for replication. There will be no restrictions on clinical setting or reported clinical outcomes.

### Types of sources

This scoping review will include experimental, quasi-experimental, and other interventional study types. Furthermore, analytical observational studies, for example cohort, case-control, and cross-sectional studies, will be included. Systematic reviews without meta-analyses will not be eligible but will be used to identify earlier studies which may be eligible. Meta-analyses which synthesise prior data to propose new comparative standards will be eligible. However, case reports, opinion pieces, editorials, and qualitative studies will be excluded.

### Search strategy

The search strategy will focus exclusively on published studies. Initially, a preliminary Ovid MEDLINE search was conducted to identify relevant articles. The keywords found in the titles and abstracts of these articles, along with the subject terms used to describe them, were utilised to develop a comprehensive search strategy for Ovid MEDLINE (see Additional File 2: Table 1). One reviewer will perform all database searches. Additionally, two librarians were consulted to enhance the robustness of the search strategy, and all team members reviewed the results of preliminary searches to ensure appropriate results were being returned, without omission of key papers. This strategy will include subject terms, which will be tailored to each database’s controlled vocabulary, and free-text terms, which will remain consistent across all databases. The electronic databases to be searched for the scoping review include Ovid MEDLINE, Ovid Embase, Scopus, EBSCOhost CINAHL, Web of Science, Cochrane Library, and IEEE Xplore. Grey literature sources will not be included in this scoping review.

Studies published in English will be considered to ensure methodologies are accurately extracted in sufficient detail for replication and to manage the volume of eligible studies.

### Study/source of evidence selection

Following the search, all identified citations will be exported into Zotero 7.0.11 (Corporation for Digital Scholarship and Roy Rosenzweig Center for History and New Media, VA, USA). Details of potentially relevant records will be uploaded to Covidence (Association for Computing Machinery, NY, USA), which will automatically identify duplicates.

Two independent reviewers will then screen the titles and abstracts to determine if they meet the eligibility criteria for the review. Full texts of records not excluded at title/abstract screening will be thoroughly evaluated against the eligibility criteria. Reasons for excluding records that do not meet the eligibility criteria will be documented and reported in the scoping review. Any disagreements between reviewers during the study selection process will be resolved through discussion and/or consultation with a third reviewer.

The search results will be fully reported in the final scoping review and presented in a PRISMA flow diagram [26].

### Data extraction

Two independent reviewers will extract data from the papers included in the scoping review using a tool they developed based on the research questions. The extracted data will cover the following:

- Study details: Author, publication year, data collection year, country, and study design
- Population demographics: Age, gender, ethnicity, medical conditions, and nutritional status including anthropometry
- Imaging modality: CT, MRI, PET, ultrasonography/elastography, and DXA
- Body composition outcomes reported (such as method, instrument, software, units)
- Details of methodology used to establish proposed comparative standards: E.g. optimal stratification (based on various outcomes), ROC analysis, sensitivity/specificity, clustering, AI-based models, and percentile-based methods
- Details of validation methodology, e.g. internal/external validation and reproducibility tests
- Comparative standards
- Outcomes reported in relation to the comparative standards, including, but not limited to, malnutrition, survival, and disease progression
- Associations/predictive value, etc. of reported outcomes in relation to the comparative standards

The data extraction tool is adapted from the JBI methodology guidelines for scoping reviews [25]. A draft data extraction table is provided in Additional File 3: Table 2. The tool will be piloted on 10 records and refined if necessary before full data extraction, after which it will remain standardised for all studies. Any disagreements between reviewers will be resolved through discussion and/or consultation with a third reviewer. Authors of papers will be contacted to request missing or additional data if necessary. Any modifications to the protocol will be detailed in the scoping review report, and the registration will be updated.

### Data analysis and presentation

In accordance with the review question, the extracted data will be displayed in tabular and/or diagrammatic form. In keeping with the goal of mapping the methodologies utilised to establish and validate comparative standards in BCI, as well as the comparative standards and evaluated outcomes, a narrative summary will be included with the tabulated and/or charted data. The type and extent of the evidence found in the scoping review will determine how the data are analysed and presented, and it may be modified accordingly.

### Assessment of methodological quality

Formal quality appraisal of included studies will not be undertaken. This aligns with JBI guidance for scoping reviews, as the intent is to map the methodologies used to establish and validate comparative standards in BCI, rather than to evaluate study quality. However, the narrative description of the included studies will include critical appraisal of the strengths and weaknesses, particularly in relation to transparency in reporting of methodology, and overall patterns of limitations in the evidence will be discussed.

## Discussion

This study will review the literature on methodologies used to establish and validate comparative standards for BCI, map the proposed comparative standards, and explore their association with clinical outcomes within the included studies. The results of the scoping review will be examined to encapsulate the current evidence on the subject and pinpoint areas where further research is necessary.

## Supplementary Information

Additional file 1. PRISMA-P 2015 Checklist.

Additional file 2. Table 1: Search strategy.

Additional file 3. Table 2: Data extraction instrument.

## Data Availability

No datasets were generated or analysed during the current study.

## Abbreviations

BCA: Body composition analysis
BCI: Body composition imaging
OSF: Open Science Framework
BIA: Bioelectrical impedance analysis
CT: Computed tomography
MRI: Magnetic resonance imaging
PET: Positron emission tomography
DXA: Dual X-ray absorptiometry
ROC: Receiver operating characteristic
AI: Artificial intelligence
PRISMA: Preferred Reporting Items for Systematic reviews and Meta-Analyses
