# Supplementary material for: Methodologies for establishing and validating cut-points and comparative standards in medical imaging-based body composition analysis: a scoping review protocol
Source: Syst Rev. 2026 Apr 10;15:169. doi: 10.1186/s13643-026-03096-y (PMC13191994; doi:10.1186/s13643-026-03096-y)
Supplement: Supplementary file 2 — Additional file 2. Table 1: Search strategy. [file 13643_2026_3096_MOESM2_ESM.docx]

## Additional File 2

## Table 1: Search strategy

MEDLINE (Ovid)

Search conducted on 25/04/2025

| # | Query | Results (25 Apr 2025) |
| --- | --- | --- |
| 1 | (sarcopeni* or body composition or fat mass or fat*free mass or muscle wasting or fat wasting or muscle loss or muscle mass or muscle volume or muscle area or fat area or fat volume or adipose area or adipose volume or adipose mass or adipose tissue area or adipose tissue volume or adipose tissue mass or muscle cross*sectional area or fat cross*sectional area or adipose cross*sectional area or adipose tissue cross*sectional area or cachexi* or cachec* or Myopeni* or myosteato* or Osteopen* or osteoporo* or bone mineral density or muscle* attenuat*).mp. [mp=title, book title, abstract, original title, name of substance word, subject heading word, floating sub-heading word, keyword heading word, organism supplementary concept word, protocol supplementary concept word, rare disease supplementary concept word, unique identifier, synonyms, population supplementary concept word, anatomy supplementary concept word] | 289,082 |
| 2 | exp Sarcopenia/ | 12,693 |
| 3 | 1 or 2 | 289,082 |
| 4 | (reference curve* or nomogram or normogram or cut*point* or cut*off value* or threshold* or reference value*).mp. [mp=title, book title, abstract, original title, name of substance word, subject heading word, floating sub-heading word, keyword heading word, organism supplementary concept word, protocol supplementary concept word, rare disease supplementary concept word, unique identifier, synonyms, population supplementary concept word, anatomy supplementary concept word] | 625,364 |
| 5 | exp Reference Values/ | 165,828 |
| 6 | exp nomograms/ | 12,296 |
| 7 | 4 or 5 or 6 | 626,672 |
| 8 | 3 and 7 | 10,694 |
| 9 | (Computed tomography or CT or PET or Positron Emission Tomography or DXA or DEXA or Dual energy x ray absorptiometr* or MRI or magnetic resonance imag* or Ultrasound or ultrasonography).mp. [mp=title, book title, abstract, original title, name of substance word, subject heading word, floating sub-heading word, keyword heading word, organism supplementary concept word, protocol supplementary concept word, rare disease supplementary concept word, unique identifier, synonyms, population supplementary concept word, anatomy supplementary concept word] | 1,954,159 |
| 10 | 8 and 9 | 3,831 |
| 11 | ((animal* or canine* or in*vitro or dog* or feline or hamster* or lamb* or mice or monkey or monkeys or mouse or murine or pig* or piglet* or porcine or primate* or rabbit* or rat* or rodent* or sheep* or veterinar*) not (human* or patient*)).ti,kf,jw. | 3,147,318 |
| 12 | (Animals/ or Models, Animal/ or Disease Models, Animal/) not Humans/ | 5,297,760 |
| 13 | 11 or 12 | 6,254,915 |
| 14 | (((sarcopeni* or body composition or fat mass or fat*free mass or muscle wasting or fat wasting or muscle loss or muscle mass or muscle volume or muscle area or fat area or fat volume or adipose area or adipose volume or adipose mass or adipose tissue area or adipose tissue volume or adipose tissue mass or muscle cross*sectional area or fat cross*sectional area or adipose cross*sectional area or adipose tissue cross*sectional area or cachexi* or cachec* or Myopeni* or myosteato* or Osteopen* or osteoporo* or bone mineral density or muscle* attenuat* or Sarcopenia) and (reference curve* or nomogram or normogram or cut*point* or cut*off value* or threshold* or reference value* or Reference Values or nomograms) and (Computed tomography or CT or PET or Positron Emission Tomography or DXA or DEXA or Dual energy x ray absorptiometr* or MRI or magnetic resonance imag* or Ultrasound or ultrasonography)) not (((animal* or canine* or in*vitro or dog* or feline or hamster* or lamb* or mice or monkey or monkeys or mouse or murine or pig* or piglet* or porcine or primate* or rabbit* or rat* or rodent* or sheep* or veterinar*) not (human* or patient*)) or ((Animals or Models, Animal or Disease Models, Animal) not Humans))).ab. or (((sarcopeni* or body composition or fat mass or fat*free mass or muscle wasting or fat wasting or muscle loss or muscle mass or muscle volume or muscle area or fat area or fat volume or adipose area or adipose volume or adipose mass or adipose tissue area or adipose tissue volume or adipose tissue mass or muscle cross*sectional area or fat cross*sectional area or adipose cross*sectional area or adipose tissue cross*sectional area or cachexi* or cachec* or Myopeni* or myosteato* or Osteopen* or osteoporo* or bone mineral density or muscle* attenuat* or Sarcopenia) and (reference curve* or nomogram or normogram or cut*point* or cut*off value* or threshold* or reference value* or Reference Values or nomograms) and (Computed tomography or CT or PET or Positron Emission Tomography or DXA or DEXA or Dual energy x ray absorptiometr* or MRI or magnetic resonance imag* or Ultrasound or ultrasonography)) not (((animal* or canine* or in*vitro or dog* or feline or hamster* or lamb* or mice or monkey or monkeys or mouse or murine or pig* or piglet* or porcine or primate* or rabbit* or rat* or rodent* or sheep* or veterinar*) not (human* or patient*)) or ((Animals or Models, Animal or Disease Models, Animal) not Humans))).ti. | 2,090 |
