# Supplementary material for: Methodologies for establishing and validating cut-points and comparative standards in medical imaging-based body composition analysis: a scoping review protocol
Source: Syst Rev. 2026 Apr 10;15:169. doi: 10.1186/s13643-026-03096-y (PMC13191994; doi:10.1186/s13643-026-03096-y)
Supplement: Supplementary file 3 — Additional file 3. Table 2: Data extraction instrument. [file 13643_2026_3096_MOESM3_ESM.docx]

## Additional File 3

## Table 2: Data extraction instrument

| Study details: author, publication year, data collection year, country, study design |  |
| --- | --- |
| Population demographics: age, gender, ethnicity, medical conditions, nutritional status incl. anthropometry |  |
| Imaging modality: CT, MRI, PET, ultrasonography/elastography, DXA |  |
| Body composition outcomes reported (method, instrument, software, units, etc.) |  |
| Details of methodology used to establish proposed cut-points: e.g., optimal stratification (based on various outcomes), ROC analysis, clustering, AI-based models, percentile-based methods |  |
| Details of validation methodology: internal/external validation, reproducibility tests, etc.. |  |
| Cut points or norms for fat, muscle, and bone |  |
| Outcomes/assocation reported in relation to the cut-points/norms: malnutrition, survival, disease progression, etc.. |  |
| Predictive value/diagnostic accuracy etc. of reported outcomes in relation to the proposed cut-points or norms |  |
